# Colon cancer diagnosis by means of explainable deep learning

**DOI:** 10.1038/s41598-024-63659-8

**Published:** 2024-07-03

**Authors:** Marcello Di Giammarco, Fabio Martinelli, Antonella Santone, Mario Cesarelli, Francesco Mercaldo

**Affiliations:** 1 Department of Medicine and Health Sciences “Vincenzo Tiberio”, University of Molise, Campobasso, Italy; 2 Institute for Informatics and Telematics, National Research Council of Italy (CNR), Pisa, Italy; 3 Department of Information Engineering, University of Pisa, Pisa, Italy; 4 Department of Engineering, University of Sannio, Benevento, Italy

**Keywords:** Colon, Cancer, Deep learning, Machine learning, Explainability, Artificial intelligence, Biomedical engineering, Cancer screening, Cancer, Engineering

## Abstract

Early detection of the adenocarcinoma cancer in colon tissue by means of explainable deep learning, by classifying histological images and providing visual explainability on model prediction. Considering that in recent years, deep learning techniques have emerged as powerful techniques in medical image analysis, offering unprecedented accuracy and efficiency, in this paper we propose a method to automatically detect the presence of cancerous cells in colon tissue images. Various deep learning architectures are considered, with the aim of considering the best one in terms of quantitative and qualitative results. As a matter of fact, we consider qualitative results by taking into account the so-called prediction explainability, by providing a way to highlight on the tissue images the areas that from the model point of view are related to the presence of colon cancer. The experimental analysis, performed on 10,000 colon issue images, showed the effectiveness of the proposed method by obtaining an accuracy equal to 0.99. The experimental analysis shows that the proposed method can be successfully exploited for colon cancer detection and localisation from tissue images.

## Introduction

Colon cancer, also known as colorectal cancer, is a type of cancer that begins in the cells of the colon, which is a part of the large intestine. It typically starts as small, noncancerous clumps of cells called adenomatous polyps. Over time, some of these polyps can become cancerous^1^.

Colon cancer is one of the most common forms of cancer worldwide. The American Cancer Society (https://www.cancer.org/cancer/types/colon-rectal-cancer/about/key-statistics.html) estimates for the number of colorectal cancers in the United States for 2024 are: About 106,590 new cases of colon cancer (54,210 in men and 52,380 in women). Risk factors for developing colon cancer include age, family history of colorectal cancer, personal history of colorectal polyps or inflammatory bowel disease, certain genetic conditions, a diet high in red or processed meats, lack of physical activity, obesity, smoking, and heavy alcohol use.

Symptoms of colon cancer may include changes in bowel habits, persistent abdominal discomfort, unexplained weight loss, fatigue, and rectal bleeding. However, in the early stages, colon cancer may not cause noticeable symptoms, making regular screening important for early detection.

Screening for colon cancer often involves tests such as colonoscopy, sigmoidoscopy, and fecal occult blood tests. Early detection^2^ is crucial in terms of reducing mortality rates and successful treatment. Early-stage cancers are typically smaller and confined to the inner layers of the colon, making them more amenable to curative treatment options such as surgery, radiation therapy, and chemotherapy. Furthermore, early-stage colon cancer may require less aggressive treatment compared to advanced-stage disease.

Adopting a healthy lifestyle, including a balanced diet, regular exercise, and avoiding known risk factors, can contribute to the prevention of colon cancer. Regular screenings are especially important for individuals with risk factors or those over the age of 50, as the risk of developing colon cancer increases with age.

Diagnosing colon cancer typically involves a combination of medical history evaluation, physical examination, and various diagnostic tests. The common method used for diagnosing colon cancer is represented by colonoscopy. During this procedure, a flexible tube with a camera on the end (i.e., the colonoscope) is inserted through the rectum to examine the entire colon. If polyps or suspicious areas are found, the doctor may take tissue samples (biopsies) for further examination under a microscope. Thus, if abnormal tissue is found during a colonoscopy or other imaging tests, a biopsy may be performed to analyze the cells under a microscope and confirm the presence of cancer.

The analysis of the biopsy is a time-consuming process performed by biologists and, for this reason, can produce misdiagnosis whether it is not performed by adequately trained medical personnel^3^.

For these reasons, in this paper, we propose a method aimed at detecting whether there is the presence of cancer in colon medical images. In particular, from the bioimages point of view, images obtained from colonoscopy are exploited, while in order to build a model aimed at discriminating between bioimages related to patients affected by colon cancer and healthy ones, we consider deep learning^4,5^, with particular regard to convolutional neural networks (CNN). While in the state-of-the-art literature, there are several proposals aimed at detecting cancer from bioimages exploiting deep learning, in the real-world practical clinic these methods are not adopted due to the lack of explainability. This is particularly important because many artificial intelligence models, such as deep neural networks, are often considered “black boxes” that make complex decisions without easily understandable reasoning. This is the reason why introduce, in the proposed method, the possibility to provide a visual explanation behind the model prediction, thus introducing explainable AI i.e., the capability of artificial intelligence systems to provide understandable and transparent explanations for their decisions and actions^6^. The visual explanation represents the research gap in the diagnostic field; so in this work, we dedicate particular attention to all qualitative aspects , applying different explainable AI techniques and, through similarity indices, try to “quantize” the qualitative results.

The paper proceeds as follows: in the next section a review of the state-of-the-art of adenocarcinoma deep learning detection; in “The method” we present the proposed method for the explainable detection of colon cancer from bioimages obtained from colonoscopy; in “Experimental analysis and results” we exploit the results and discuss them; and, finally, in the last section we present the conclusion and future research on this topic.

## Related work

In this section, we present a review of the state-of-the-art research on the adoption of deep learning in the context of adenocarcinoma detection followed by a reasoned discussion.

Authors in^7^ applied to the CRC-5000, nct-crc-he-100k and merged datasets on the ResNEt network obtaining 96.77%, 99.76% and 99.98% for the three publicly available datasets, respectively. Moreover, they tested their training strategy and models on the CRC-5000, nct-crc-he-100k and Warwick datasets. Respective accuracy rates of 98.66%, 99.12% and 78.39% were achieved by SegNet. However, the authors focused only on quantitative results and didn’t take into account the qualitative aspects. In the work of Musad et al.^8^, authors applied to CNNs architecture the same dataset, including the three folders of lung tissue and provided a 5-classes distinction obtaining 96.33% in accuracy. They applied the following techniques: wavelet and DFT (Discrete Fourier Transform) for the pre-processing steps and non-specified fully connected CNN for the classification task. From a medical point of view, analyzing lung and colon histological images into the same classification is not used in practice. Also in^9^, the same dataset was used. In this paper, authors reached 99% and 100%applying the DL network, suh as VGG16, VGG19, MobileNet, DenseNet169, and DenseNet201. These good performances were reproducible, but remain only from the metrics point of view. An interesting approach was explained in^10^. In their approach, the image classes (LC25000 datset) were trained from scratch with the DarkNet-19 model, and two optimization algorithms (Equilibrium and Manta Ray Foraging) and combined with the Support Vector Machine (SVM) method provided a performance of 99.69%. In the paper of Mehmood^11^, the LC250000 dataset was considered by a modified version of the AlexNet network for training and testing. In this way, performances reach 98.4% for a 5-way classification. Also in this, the reasons of mixing the lung and colon histological images are not clear and it is missed the visual explanation of these results. Three CNNs were trained and tested in^12^. They used three pre-trained CNN models, which are ShuffleNet V2, GoogLeNet, and ResNet18 also one simple customized CNN model. ShuffleNet V2 was the best model used to classify colon data, it gives 99.87% accuracy with the fastest training times of 1202.3 seconds. In the work of Sakr^13^, the input histopathological images (LC25000 dataset) were normalized before feeding them into their CNN model, and then colon cancer detection was performed. The result analysis demonstrates that their proposed deep model for colon cancer detection provides a higher accuracy of 99.50%, Bukhari and his collegues^14^ provided two colon images datasets: LC250000 and Colorectal Adenocarcinoma Gland (CRAG) Dataset. In their study, three variants of CNN (ResNet-18, ResNet-34 and ResNet-50) have been employed to evaluate the images.The accuracy (93.91%) of ResNet-50 was the highest which is followed by ResNet-30 and ResNet-18 with the accuracy of 93.04% each. Last work^15^ processed the LC25000 dataset. A shallow neural network architecture was used to classify the histopathological slides into squamous cell carcinomas, adenocarcinomas and benign for the lung. A similar model was used to classify adenocarcinomas and benign for the colon. The diagnostic accuracy of more than 97% and 96% was recorded for lung and colon respectively.

Table 1 compares the works covered in this section with the proposed approach in this study. It shows the important findings, the used dataset, and whether or not the authors consider explainability for the localization of disease into the images.

**Table 1:** Comparison between the proposed method and the current leading methods found in the state of the art.

Authors	Dataset	Methods	Localization	Results
Hamida et al. (2021)	CRC-5000			96.77% and 98.66%
	nct-crc-he-100k	ResNet and SegNet	No	99.76% and 99.12%
	Merged datasets			99.98% and 78.39%
Masud et al. (2021)	LC250000 dataset	Fully connected CNN	No	96.33%
Talukder et al. (2022)	LC250000 dataset	VGG16, VGG19, MobileNet	No	from 99 to 100%
		DenseNet169, and DenseNet201		
Tougacar et al. (2021)	LC250000 dataset	DarkNet-19 model combined	No	99.69%
		with the Support Vector Machine		
Mehmood et al. (2022)	LC250000 dataset	AlexNet	No	98.4%
Wahid et al. (2023)	LC250000 dataset	ShuffleNet V2		
		GoogLeNe	No	99.87% (ShuffleNet V2)
		ResNet18		
Sakret al. (2022)	LC250000 dataset	Different layer number	No	99.50%
		of customized model (CN)		
Bukhari et al. (2020)	LC25000 dataset	ResNet-18		93.04%
	CRAG dataset	ResNet-34	No	93.04%
		ResNet-50		93.91%
Mangal et al. (2020)	LC25000 dataset	Shallow neural network architecture	No	96%
Our method	LC250000 dataset	ResNet 50		65%
		DenseNet	Yes (Grad-CAM	68%
		VVG19	Score-CAM	49%
		Standard_CNN	FastScore-CAM	50%
		Inception-V3	and MR-SSIM)	99%
		EfficientNet		99%
		MobileNet		99%

## The method

**Figure 1:**
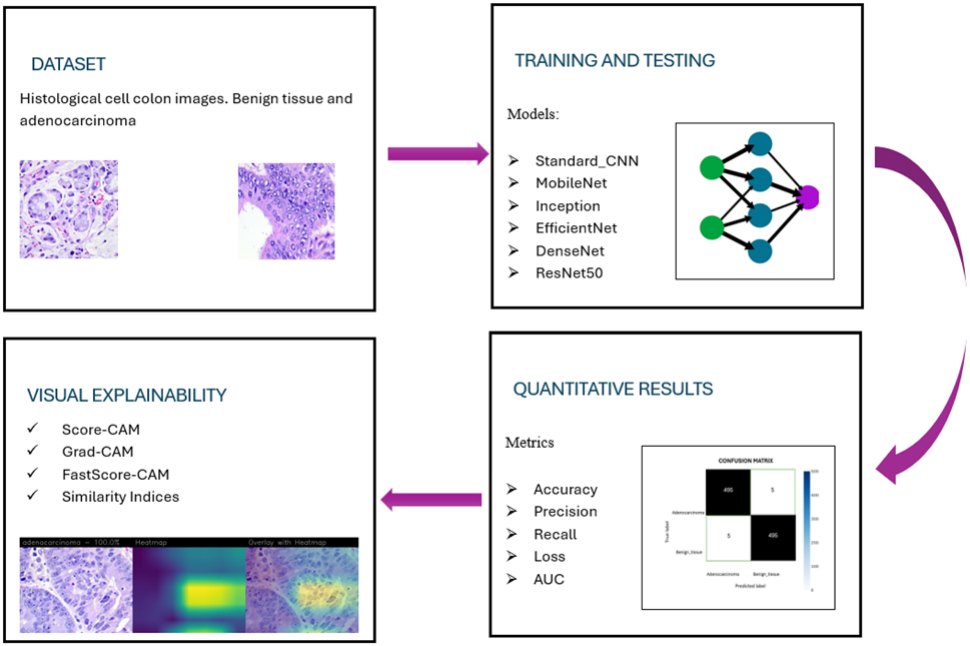
Main steps of the proposed methodology.

In this section, the proposed method for the detection of adenocarcinoma cancer coming from cell colon images is presented and shown in Fig. 1.

Starting from the first step, the dataset’s choice represents one of the fundamental steps for the problem under analysis. For the classification of adenocarcinoma, authors choose a binary classification, considering a dataset was initially composed of 500 histological images of the colon cells for each class (benign tissue and adenocarcinoma) uploaded from Kaggle dataset website, loaded by Larxel, a Senior Data Scientist working at Hospital Israelita Albert Einstein, São Paulo, State of São Paulo, Brazil (https://www.kaggle.com/andrewmvd/datasets).

**Figure 2:**
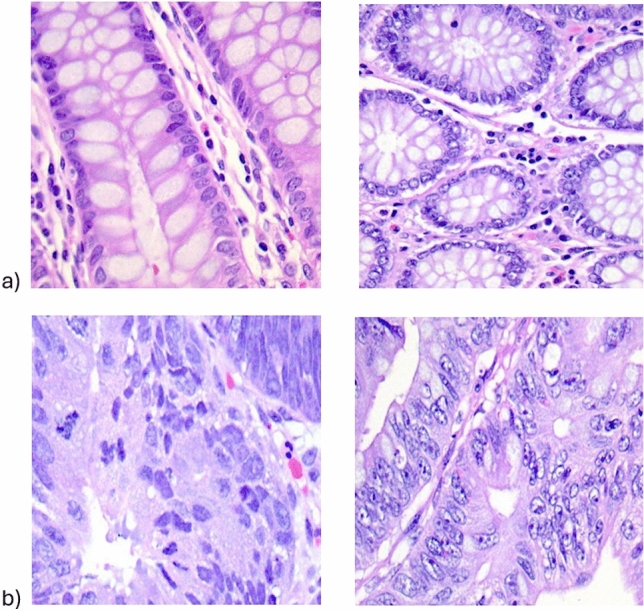
Representative images of the dataset: (**a**) benign tissue, (**b**) adenocarcinoma below.

Figure 2 shows examples of colon cell histological images: benign tissue (Fig. 2a) and adenocarcinoma cells (Fig. 2b). For the medical contest, it is relevant that the images are generated and validated by specialists. In this way, the dataset is usable and reproducible for research purposes.

The exploited dataset contains 5000 images for each class, as a data augmentation process was applied, using the Augmentor package. The applied data augmentation involves creating modified versions of the original samples by applying transformations like rotation, scaling, cropping, and flipping to increase the diversity of the data available for training. Augmentor package in Python is Compatible with Python 2 and 3 versions, and several kinds of image formats) simplifies this process by providing an easy-to-use interface for generating augmented data. Data augmentation guarantees an increase with a factor of ten of the sample images, providing the training step with a more useful and generalized dataset. Further information and methods regarding data augmentation in medical imaging contest are reported in^16–18^.

Other pre-processing techniques (for instance, denoising^19^) are not taken into account, because the dataset reports with an adequate number of samples and good resolution. Further, pre-processing steps improve the computational costs and time consumption. The following step consists of the training of CNNs and the testing of these latter. All the training and testing phases, setting the hyperparameters and the sample splitting, generate a network model (for each architecture) through which results evaluation was obtained.

In the last two steps, the results evaluated from a quantitative to qualitative point of view were presented and discussed.

Quantitative results refer to the metrics, such as accuracy, precision, recall, loss, and Area Under the Curve (AUC). These results are supported by the confusion matrix computation and the graphical representation of the accuracy epoch and loss-epoch trends.

On the other hand, a qualitative analysis is conducted to explain the models developed. Class Activation Mapping (CAM) algorithms, specifically Grad-CAM and Score-CAM, generate heatmaps on input images, improving visual explainability and localization features. In conclusion, a structural similarity analysis was applied to these heatmaps.

### CAM algorithms and visual explainability

Visual explainability for the resulting models was provided via CAM algorithms, specifically Grad-CAM and Score-CAM, as well as Structural Similarity Index Measures (SSIM).

In the context of CNNs and CAM algorithms, the selected layer for CAM is typically the final convolutional layer before the global average pooling layer or the fully connected layer in the network architecture. CAM algorithms aim to highlight the regions of an input image that contribute the most to the prediction made by the model. This is achieved by visualizing the activation maps of the final convolutional layer, which captures the spatial information learned by the network. The final convolutional layer is chosen because it retains spatial information about the input image while also abstracting high-level features learned by the network. By examining the activation maps of this layer, CAM techniques can localize the relevant features in the input image that are used by the model to make predictions. Some guidelines, during the qualitative evaluation of the models, help the data scientist to choose a better model that presents a visual explanation for medical staff. The considerations to take into account are related to:

- knowledge about to images under exam (imaging techniques, disease features, and his severity degrees);
- presence of the Region Of Interest (ROI) according to the presence of the disease;
- the shape of these ROIs, for instance in histological images, the irregularity of the patterns can be crucial;
- does not focus only on a few samples but evaluates the qualitative trends of the heatmaps for the entire sample set.

By highlighting the ROIs of the image that have contributed most to the classification; of medical content, the presence of the disease, for instance, the cancerous cells in colon images, CAM-based algorithms can enable us to understand the most discriminating feature of the images and to identify potential regions that have not yet been considered in current research, thereby directing future developments. Furthermore, concerning the pattern relevance throughout the classification process, the heatmaps are based on the VIRIDIS coloration (https://cran.r-project.org/web/packages/viridis/vignettes/intro-to-viridis.html).

In addiction, CAM algorithms and ROI analysis serve complementary roles in medical imaging analysis, with CAM providing insights into image interpretation and ROI analysis focusing on the detailed examination of specific regions within the image. While they are not directly correlated, they can be used synergistically to improve the understanding and utility of medical imaging data.

Looking in deeper detail at the two CAM algorithms: the Gradient-weighted Class Activation Mapping (Grad-CAM)^20^ which applied the back-propagation of individual class weights, highlights ROIs, considering the gradient of the pixels in the images. A different method is used by the Score-weighted Class Activation Mapping (Score-CAM)^21^ algorithm that based the heatmaps generation on the score i.e. the Channel-wise Increase of Confidence (CIC) parameter, which is used by Score-CAM to evaluate each feature map’s contribution based on the class score.

FastScore-CAM^22^ is an enhancement or optimization of the Score-CAM method. It is designed to be computationally more efficient and faster to compute compared to Score-CAM. The term “Fast” in FastScore-CAM suggests that it introduces improvements in terms of speed or efficiency, making it more suitable for real-time or large-scale applications. It aims to retain the interpretability and accuracy of the original Score-CAM while addressing potential computational bottlenecks. In summary, FastScore-CAM is a variant of Score-CAM that is designed to be faster in terms of computation while preserving the interpretability of the original method.

The Structural Similarity Index Measure (SSIM) is a widely-used metric for quantifying the similarity between two images. It was introduced by Wang^23^ in 2004. SSIM compares three aspects of images: luminance, contrast, and structure. It assesses the perceived change in structural information, which is crucial for human perception of image quality. In the DL contest, the SSIM approach was applied to improve the models’ level of explainability^24^. Conceptually, this technique “quantizes” the qualitative results coming from the overlapped heatmaps on cell colon histological images, providing values of similarity. These values indicate the degree of difference between two heatmaps created using the same model but different CAM techniques. The values of this index range from +1 to − 1, where a value of +1 denotes equality between the two images. The SSIM technique compares the images considering the differences in brightness, contrast, and potential distortions. In the proposed approach, we evaluate the qualitative model robustness, the MR-SSIM (Model Robustness SSIM) must have higher values.MR-SSIM refers to the application of the SSIM to evaluate the model robustness, so MR indicates only the final aims of the indices. This means that the comparison of two CAMs highlights the common pattern in the same image guaranteeing robustness for the classification model, and the explanation of the latter.

CAM algorithms and similarity indices introduce and explore qualitative aspects, and provide visual explainability to the AI “black box” model for medical and diagnosis point of view.

## Experimental analysis and results

This section presents the dataset that was taken into account for the obtaining of quantitative and qualitative results. The latter were reported and discussed. The dataset we exploited is freely available for research purposes and is available at the following url (https://www.kaggle.com/datasets/andrewmvd/lung-and-colon-cancer-histopathological-images/code) on the Kaggle website. This dataset considers two main directories: one refers to the lung cancer images and the other to the colon cancer images. Coherently to the topic of the paper, we consider only the colon cancer folders, which are composed of two classes: benign tissue and adenocarcinoma (i.e., a binary classification is exploited). As discussed in “The method” the dataset was augmented, generating 5.000 histological images for each class (we consider a binary classification i.e., Adenocarcinoma and Benign_tissue). For the DL classification, the dataset was divided into an 80-10-10 splitting for the training, validation, and testing sets, respectively. The splitting division of the samples is the following:

- 80% of images (8.000) to the training dataset
- 10% of images (1.000) to the validation dataset.
- 10% of images (1.000) to the testing dataset.

For the training-testing phase, seven different deep learning architectures were been considered: ResNet50^25^, DenseNet^26^, VGG19^27^, Standard_CNN^28,29^, Inception-V3^30^, EfficientNet^31^ and MobileNet^32^. The hyper-parameters are set to 50 epochs, 8 as batch, 0.0001 learning rate, and $224 \times 224 \times 3$ image size. This combination is determined by evaluating several combinations on the networks under investigation.

We exploited the binary cross-entropy as loss function. As a matter of fact, using binary cross-entropy is specifically designed for binary classification problems, making it well-suited for tasks where the output variable has only two possible outcomes. Infact, binary cross-entropy is specifically designed for two-class classification problems where each input can belong to only one class among two mutually exclusive classes. Moreover, it mathematically penalizes the distance between the predicted probability distribution and the actual distribution of the class. This is the reason why it can be considered a good choice for optimizing models to predict class probabilities.

All training and testing were performed in a working environment using an Intel Core i7 CPU with 16 GB RAM.

In Table 2 are reported the metrics of the networks in terms of accuracy, precision, recall, F-Measure, AUC, and loss.

The classification results are shown in Table 2.

**Table 2:** Metrics evaluation for tested DL models.

Architectures	Accuracy	Precision	Recall	F-Measure	AUC	Loss
ResNet 50	0.652	0.652	0.652	0.652	0.677	1.039
DenseNet	0.688	0.688	0.688	0.688	0.696	22.997
VGG19	0.499	0.499	0.499	0.499	0.5	0.697
Standard_CNN	0.5	0.5	0.88	0.5	0.5	0.693
Inception-V3	0.998	0.998	0.998	0.998	0.999	0.004
EfficientNet	0.996	0.996	0.996	0.996	0.996	0.446
MobileNet	0.999	0.999	0.999	0.999	0.998	0.045

From Table 2 two different groups of architectures were identified, based on the metrics results. The first one, which comprises the VGG19, the Standard_CNN, the ResNet50, and the DenseNet presents low results. These networks are not able to classify correctly the images, increasing the error possibility, and consequently are not reliable for the adenocarcinoma diagnosis; these networks will be excluded for further analysis.

On the other hand, the second group of CNNs, i.e. EfficientNet, MobileNet and Inception-V3 show optimal quantitative metrics, reaching almost 100% for accuracy, precision and recall. In other words, the classification applied through these architectures guarantees correct diagnosis for histological colon images. Additionally, these results confirm the author’s choice of not applying other pre-processing steps on the dataset, obtaining minor time consumption and computational cost.

**Figure 3:**
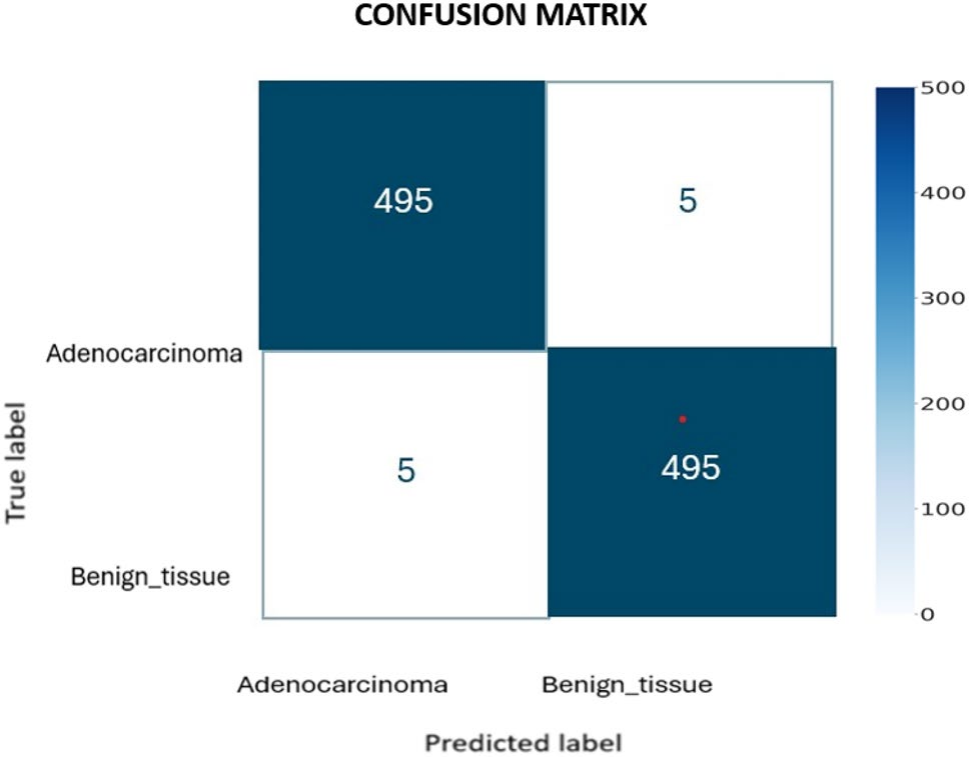
Confusion matrix of the MobileNet network.

To emphasize these results, Fig. 3 reported the confusion matrix considering the MobileNet network.

The matrix in Fig. 3 demonstrates the model’s good performance, with greater values on the first diagonal, indicating that objects classified in a specific class are properly predicted in that class.

**Figure 4:**
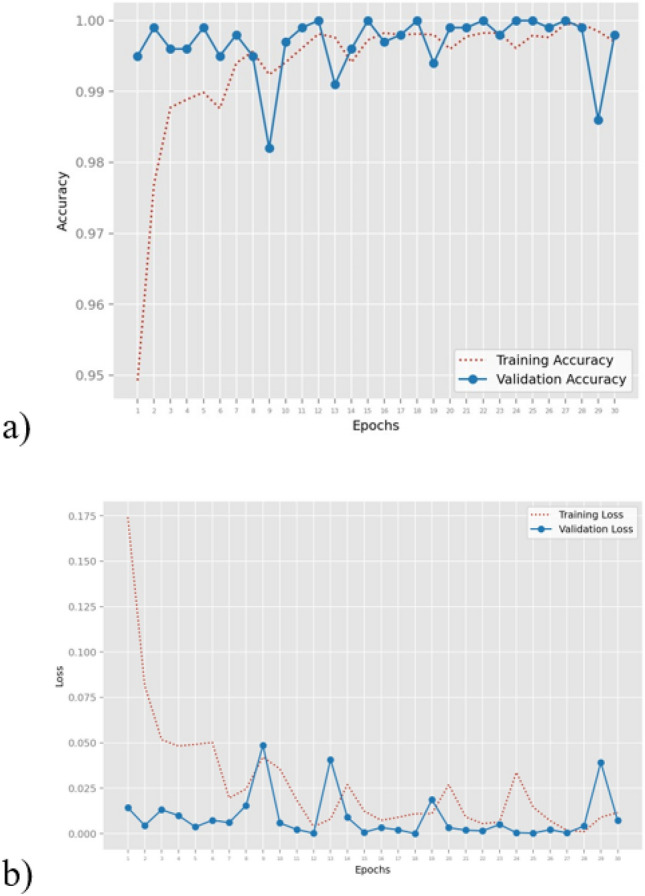
Epoch trends of the MobileNet network: (**a**) epoch-accuracy plot; (**b**) epoch-loss plot.

In Fig. 4 is shown for MobileNet network the epoch-accuracy and epoch-loss trends.

Good training phase results are shown in Fig. 4a, with a minor decline observed during the validation phase (blue line). The training accuracy trend (red dotted line) demonstrates that the MobileNet model was able to identify the differences between images belonging to distinct classes. Figure 4 illustrates the opposite behavior that is acquired from the (training and testing) loss, providing more evidence that the model is correctly learning the distinctions between cells from benign tissue and those from adenocarcinoma. From these trends, it is possible to observe the convergence of loss, that is when the loss curve converges to a relatively stable value over epochs. This suggests that the model has learned the underlying patterns in the data and is not overfitting or underfitting. From both plots, it is present the alignment of training and validation curves. Indeed, ideally, the training and validation curves should follow a similar trend. This indicates that the model is generalizing well to unseen data.

### Qualitative analysis

In this sub-section, the qualitative results were illustrated and discussed.

For these results, the quantitative approach is not valid because the qualitative aspect is not related to a quantified measure, but is based on the explanation conducted directly on the heatmaps overlapped on input images. So, to perform this evaluation, we have some guidelines presented in “The method”. After the heatmap generation on the three considered models and the three CAM algorithms; three different results were obtained.

Inception-V3 cannot able to generate heatmaps; this behavior is typical when a model does not recognize any common patterns in the images. From the qualitative point of view, this model is not able for a visual explanation.

**Figure 5:**
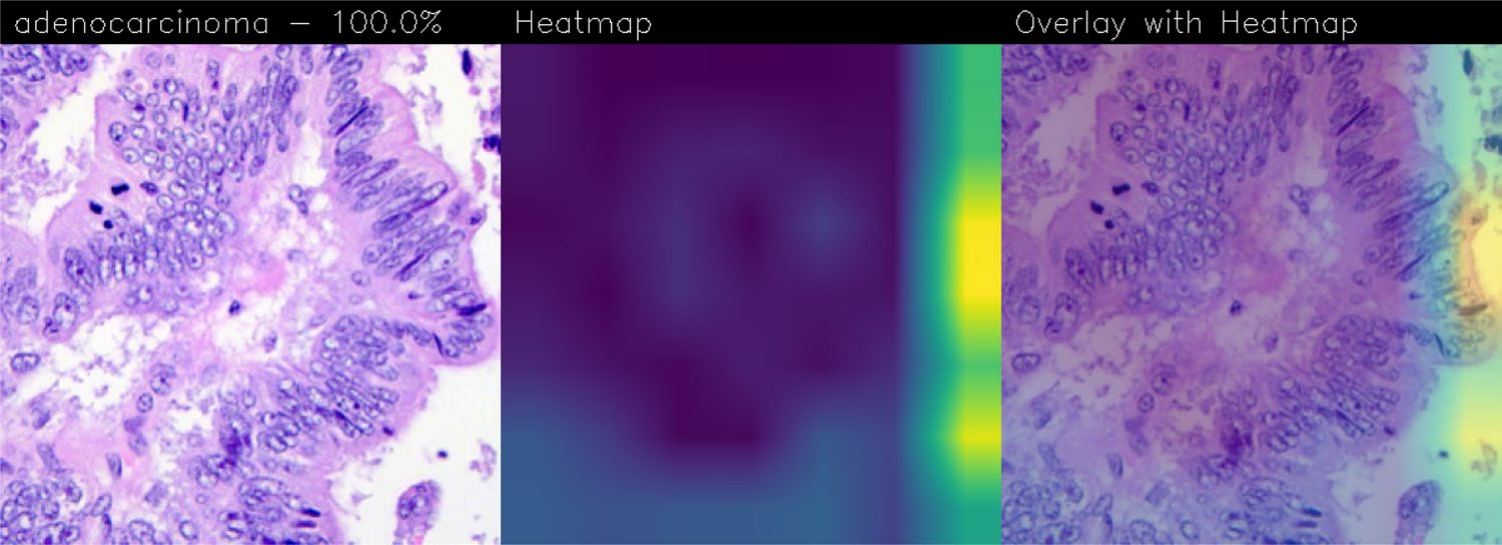
Grad-CAM of the EfficientNet model.

EfficientNet model generates heatmaps, but by analyzing the entire sets of samples, it is possible to observe that all the highlighted heatmaps are identical, and in this case are focused on the right side as shown in Fig. 5.

This behavior occurs when the models reveal a single pattern and repeat the same heatmaps for all the samples, not considering the variations in the input image. The same heatmaps appear also in the Score-CAM and FastScore-CAM. From a general point of view, CAM algorithms rely on the learned feature representations of the neural network model, which may not always align perfectly with the subtle visual cues associated with the presence of disease in medical images. If the model architecture or the training data does not adequately capture the relevant features indicative of the disease, the CAM-generated heatmaps may not accurately highlight the areas of interest. Considering that all the networks are trained and tested with the same dataset and with the optimal hyper-parameters combination, the main differences regarding the network architecture and the corresponding generated model. Moreover, in medical imaging classification, it is important to remember that the same networks work with good performances for all the medical images or all diseases. Consequentially, for each dataset and each classification task, an accurate comparison of CNNs is necessary.

**Figure 6:**
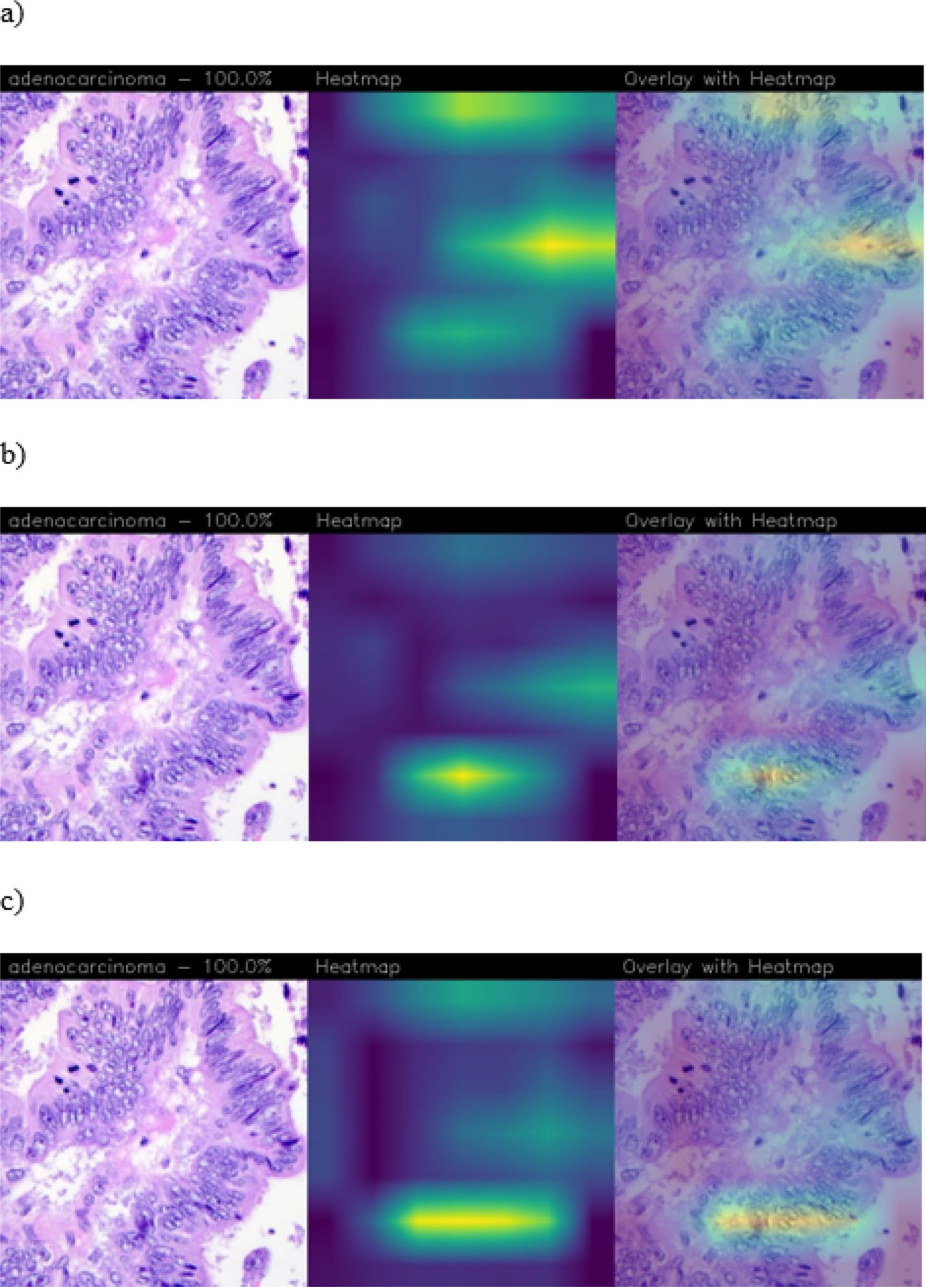
CAMs for MobileNet network of the same sample: (**a**) Grad-CAM, (**b**) Score-CAM, (**c**) FastScore-CAM.

For MobileNet, the obtained heatmaps are related to the presence of the ROIs corresponding to the presence of the disease, i.e. adenocarcinoma cell clusters, as shown in Fig. 6.

In Fig. 6 were reported the heatmaps of the same samples for the three applied CAMs. The CAMs highlight three areas: in the upper, on the right side and in the bottom. varying the CAMs algorithm varies the intensity related to these common patterns, and these are referred to the presence of tumoral cell clusters. In such way, the heatmaps provide visual explainability and localization of the disease presence, improving reliability, trustworthiness and credibility from a medical point of view.

Furthermore, the authors attempt to quantify the qualitative results and improve the model’s robustness by introducing the MR-SSIM. Table 3 displays the average similarity value among Grad-CAM, Score-CAM and FastScore-CAM heatmaps for each class, considering a couple of heatmap sets and obtaining the three possible combinations.

Table 3 compares heatmaps activated by Grad-CAM, Score-CAM, and FastScore-CAM algorithms on the same model, i.e., MobileNet. The MR-SSIM indices report 0.79 for the Grad-CAM/Score-CAM comparison and 0.76 for Score-CAM/FastScore-CAM as higher values. This means that the heatmaps produced by two distinct CAMs are highly similar, identifying the same locations with little changes in intensity.

**Table 3:** MR-SSIM for the heatmaps sets.

Similarity index for MobileNet model		
Heatmaps couple sets	Classes	MR-SSIM
Grad-CAM/Score-CAM	Benign_tissue	0.797
	Adenocarcinoma	0.790
Grad-CAM/FastScore-CAM	Benign_tissue	0.691
	Adenocarcinoma	0.743
Score-CAM/FastScore-CAM	Benign_tissue	0.762
	Adenocarcinoma	0.746

When applying the SSIM to different CAM algorithms in adenocarcinoma biopsy images, the objective is to assess how well these algorithms highlight ROIs indicative of adenocarcinoma presence while preserving the structural details present in the original biopsy images. High values enhance os SSIM between two CAMs means that different CAM algorithms highlight the same areas (ROIs), improving in this way the visual explanation.

## Conclusion and future works

In this paper, we designed and experimented with an automated approach for detecting adenocarcinoma in the colon tract by using histological images of colon cells. The results show that the deployed CNNs, specifically MobileNet, Inception-V3, and EfficientNet architectures, report optimal quantitative performances in terms of accuracy, precision, and recall (99% in each).

This work focuses on the qualitative aspects of tested models, using CAM algorithms to the visual localization of relevant and common patterns in the images related to the network classification and the cancer cell clusters in colon tissue. Furthermore, the use of three distinguished CAMs, i.e. Grad-CAM, Score-CAM, and FastScore-CAM, united to the index similarity; improves the reliability and the trustworthiness of AI in healthcare. This indicates that while deep learning prediction does not replace human decisions, it does aid in the consultation process during the diagnostic procedure.

Future studies will focus on different types of colon cancer generated by other imaging techniques (CT, MRI, echography, etc.) and combine them into an assembled DL model. Moreover, we will design a set of adversarial machine learning related to data poisoning to evaluate the DL bioimages classification resilience to these techniques. As a future work, more comparative evaluations can be performed after applying capsule network-based methods since they can keep spatial relationships of learned features and have been used recently in various works^33,34^ and transformer-based approaches since they can obtain global information effectively^35^.

## Data Availability

The datasets generated and/or analysed during the current study are available in the “Lung and Colon Cancer” repository on Kaggle website, at the following link: https://www.kaggle.com/datasets/biplobdey/lung-and-colon-cancer.
